# Supplementary material for: Universal paramyxovirus vaccine design by stabilizing regions involved in structural transformation of the fusion protein
Source: Nat Commun. 2024 May 31;15:4629. doi: 10.1038/s41467-024-48059-w (PMC11143371; doi:10.1038/s41467-024-48059-w)
Supplement: Supplementary file 1 — Supplementary Information [file 41467_2024_48059_MOESM1_ESM.pdf]

Supplemental information

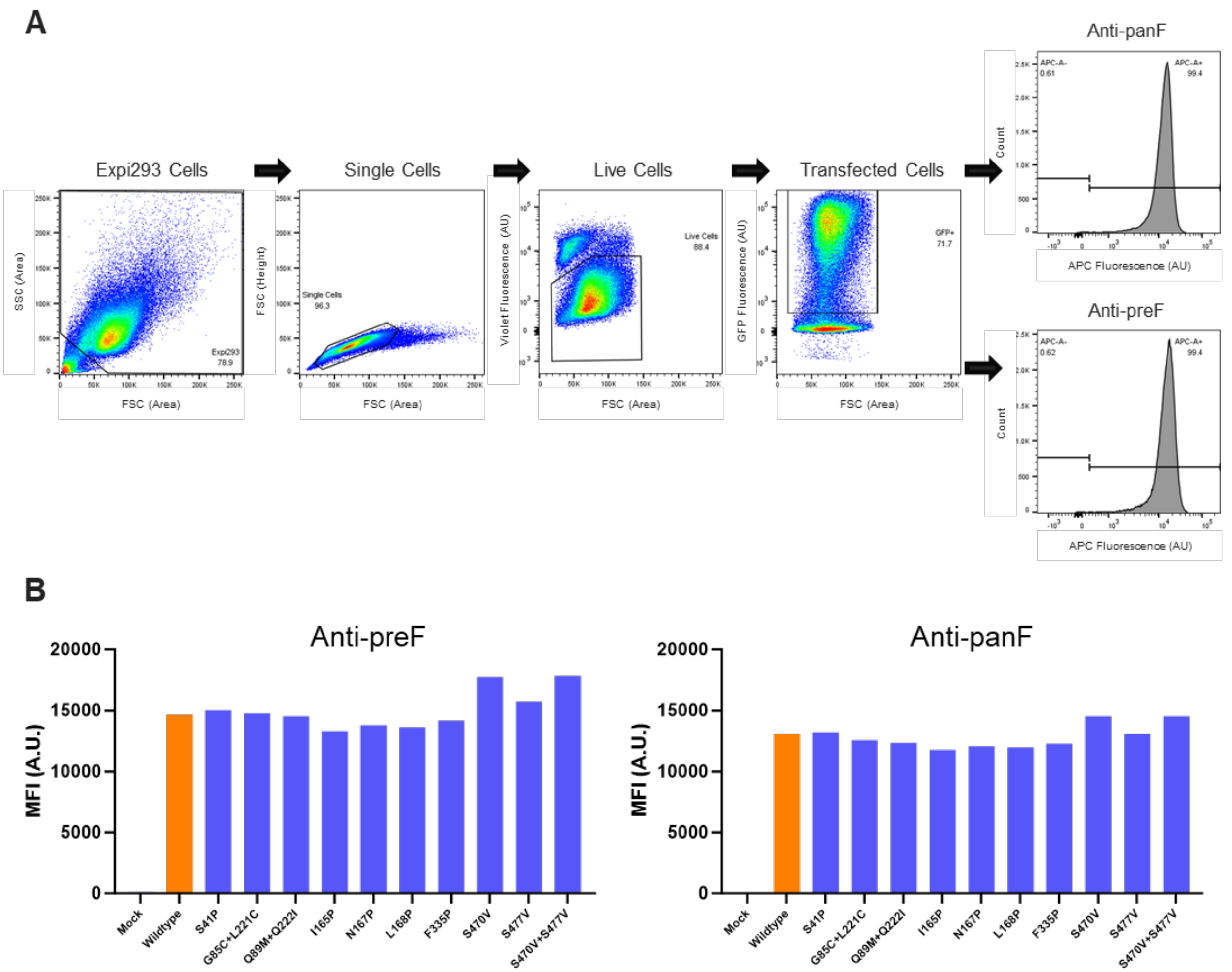

Supplementary Figure 1. Quantification of full-length RV3 F expression using FACS and preF-specific antibody PIA174 on cells transiently transfected with F plasmids of Figure 3 and stained 24 h later. (A) Gating strategy. (B) Mean fluorescence intensity (MFI) of preF- (left panel) and panF-positive (right panel) cells.

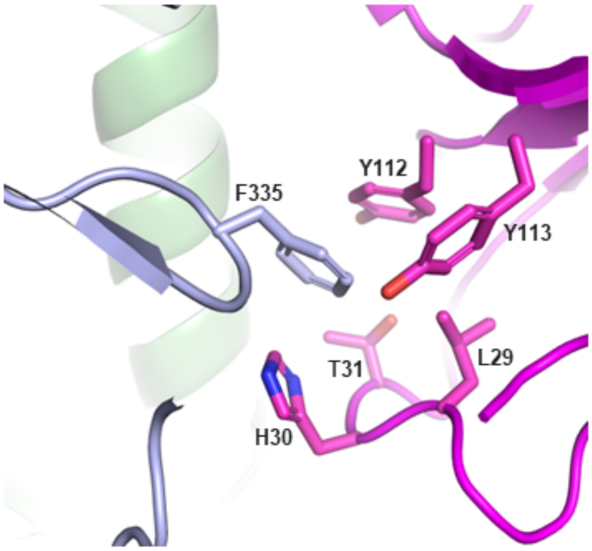

Supplementary Figure 2. Cartoon and stick representation of the interface between F335 in RV3 F2 (blue) and sdAb 4C03 (magenta). The hydrophobic pocket where F335 docks on 4C03 is shown as stick representation.

A

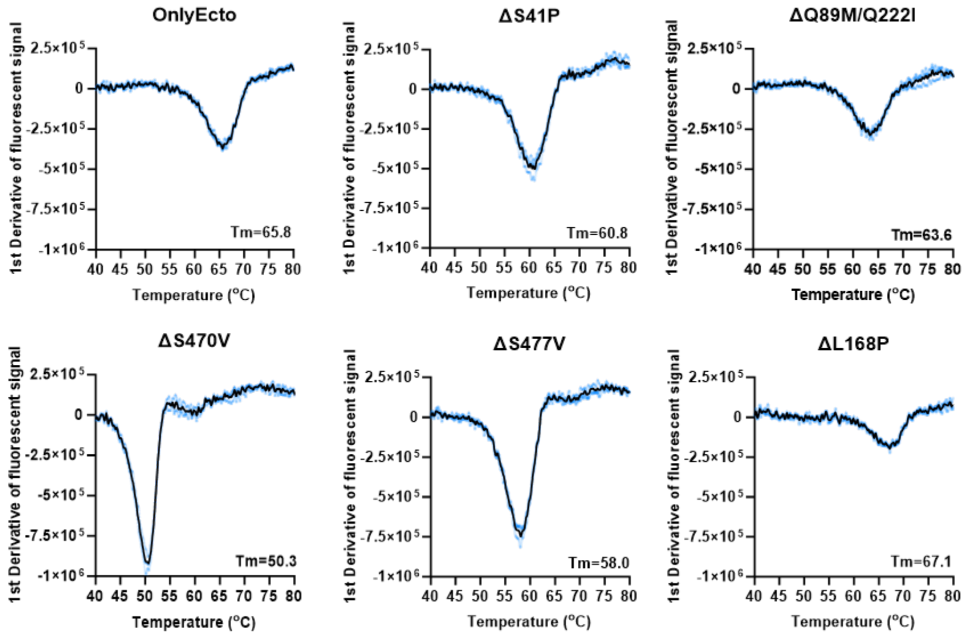

B

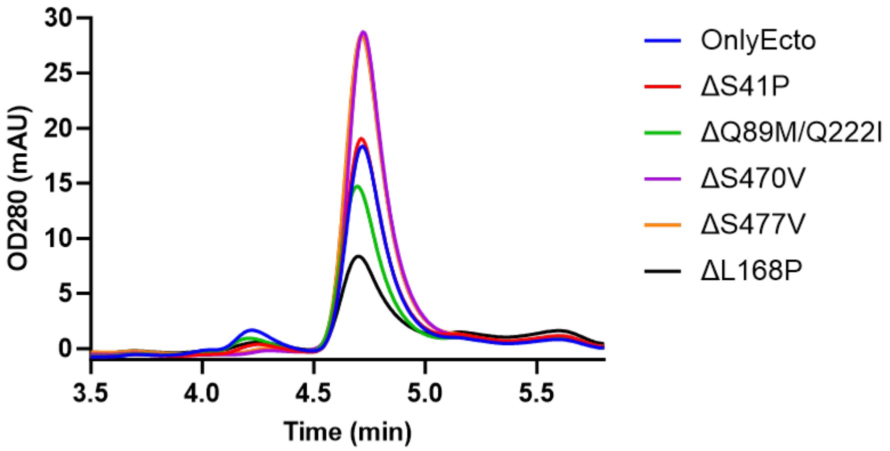

Supplementary Figure 3. RV3 preF-stabilizing substitutions. (**A**) DSF on indicated F variants in crude cell supernatant. (**B**) Analytical SEC on F variants from (A).

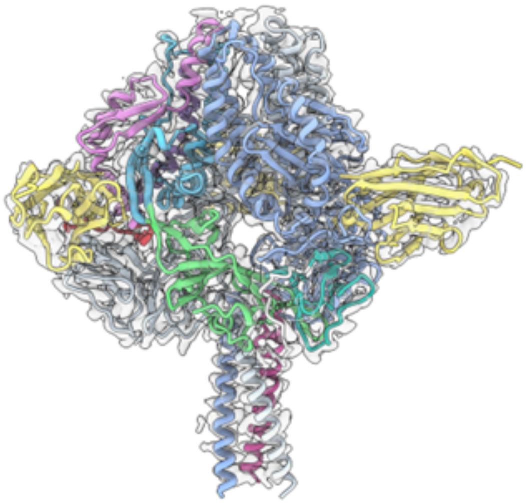

Supplementary Figure 4. Map and structure of EctoOnly2P in complex with sdAb 4C06. The structure is depicted as cartoon and colored according to domain as in Fig 1A; 4C06 is shown in yellow. The map is shown at 80% transparency.

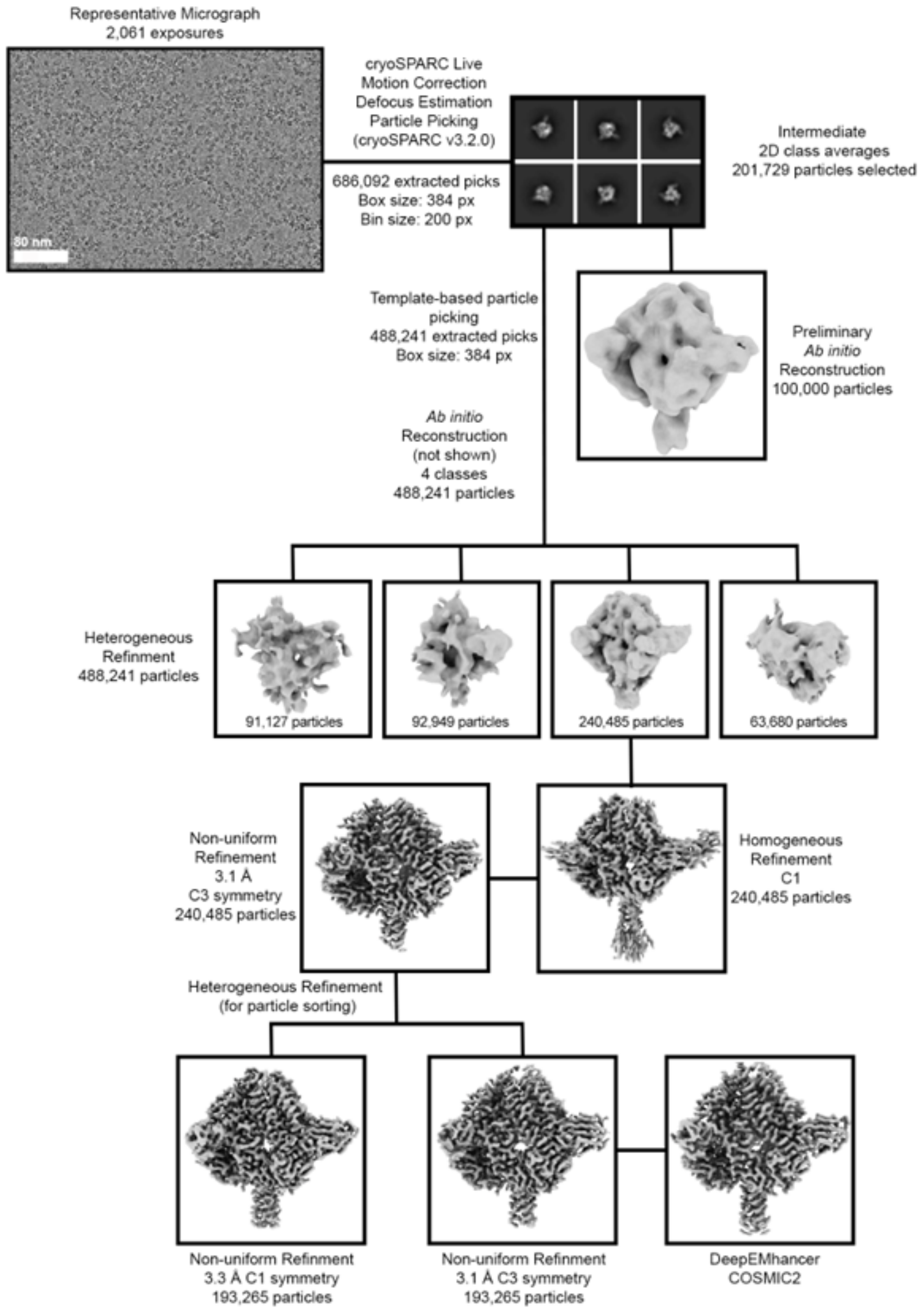

Supplementary Figure 5. Cryo-EM data processing workflow. Flowchart outlining cryo-EM data processing of RV3 preF with VHH 4C06 bound. Additional information can be found in the Methods section under "Cryo-EM data processing and model building."

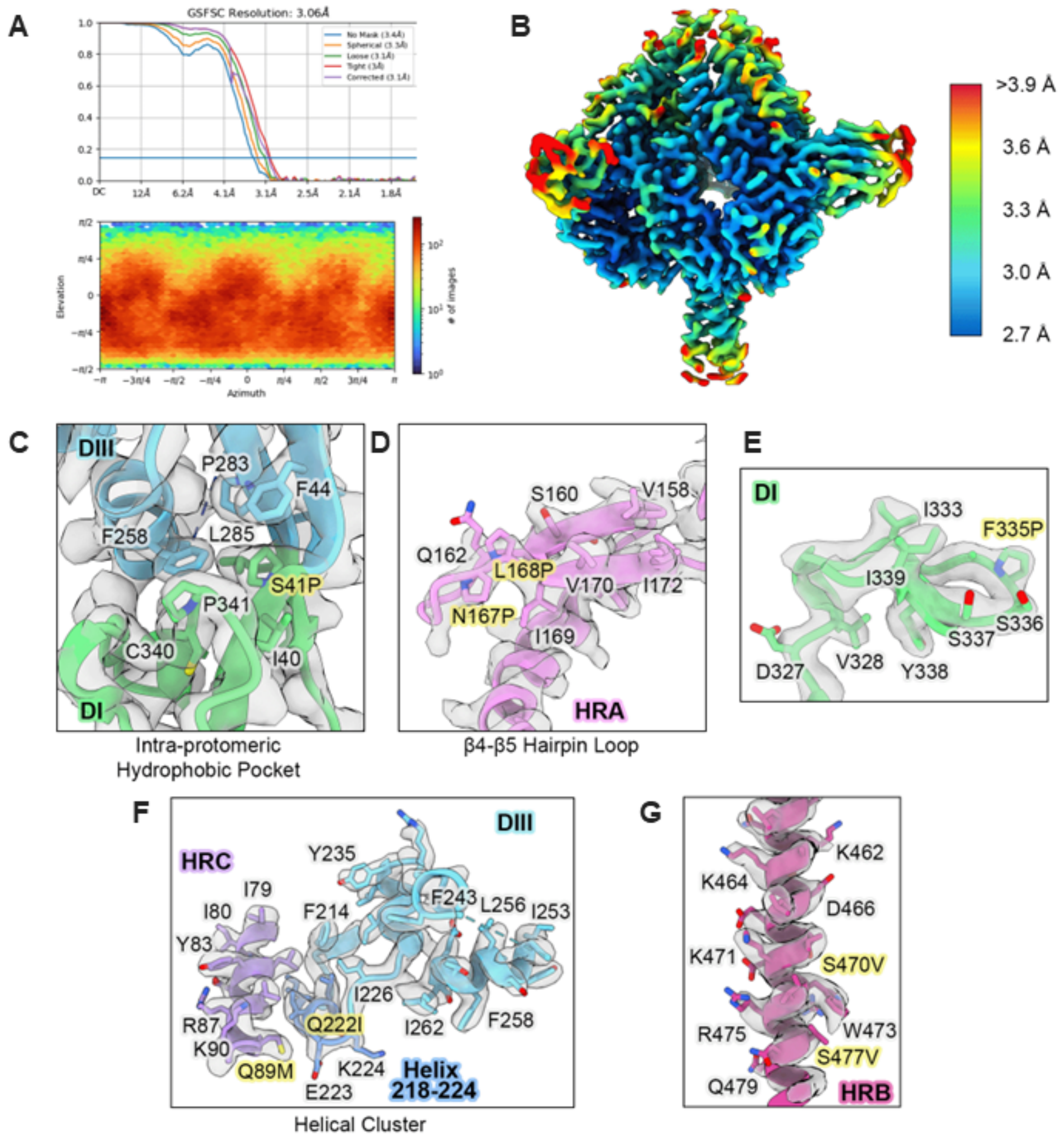

Supplementary Figure 6. Cryo-EM structure validation. (**A**) FSC curve and distribution plot for the C3 RV3 preF/4C06 structure, generated in cryoSPARC v3.2.0 [58]. (**B**) Local resolution, indicated by color, of the C3 RV3 preF/4C06 reconstruction. (**C**) Detailed view of the S41P substitution and surrounding residues within the intra-protomeric hydrophobic pocket. Cryo-EM map is shown in 70% transparent silver and the model is shown as a cartoon with selected residues shown as sticks. DI: domain I, colored green. DIII: domain III, colored light blue. Nitrogen atoms are colored blue, and sulfur atoms are colored yellow. (**D**) Detailed view of the N167P + L168P substitutions

and surrounding residues within and near the β4-β5 hairpin loop. Cryo-EM map is shown in 70% transparent silver and the model is shown as a cartoon with selected residues shown as sticks. HRA: heptad repeat A, colored pink. Nitrogen atoms are colored blue, and oxygen atoms are colored red. (**E**) Detailed view of the S470V + S477V substitutions and surrounding residues. Cryo-EM map is shown in 70% transparent silver and the model is shown as a cartoon with selected residues shown as sticks. HRB: heptad repeat B, colored magenta. Nitrogen atoms are colored blue, and oxygen atoms are colored red. (**F**) Detailed view of the Q89M + Q222I substitutions and surrounding residues within and near the helical cluster in the DIII core. Helix 218-224 is newly resolved in the RV3 preF model. Cryo-EM map is shown in 70% transparent silver and the model is shown as a cartoon with selected residues shown as sticks. HRC: heptad repeat C, colored purple. DIII: domain III, colored light blue. Helix 218-224 within DIII is colored cornflower blue. Nitrogen atoms are colored blue, oxygen atoms are colored red, and sulfur atoms are colored yellow. (**G**) Detailed view of the F335P substitution and surrounding residues within the surface exposed β hairpin within DI. Cryo-EM map is shown in 70% transparent silver and the model is shown as a cartoon with selected residues shown as sticks. DI: domain I, colored green. Nitrogen atoms are colored blue, and oxygen atoms are colored red.

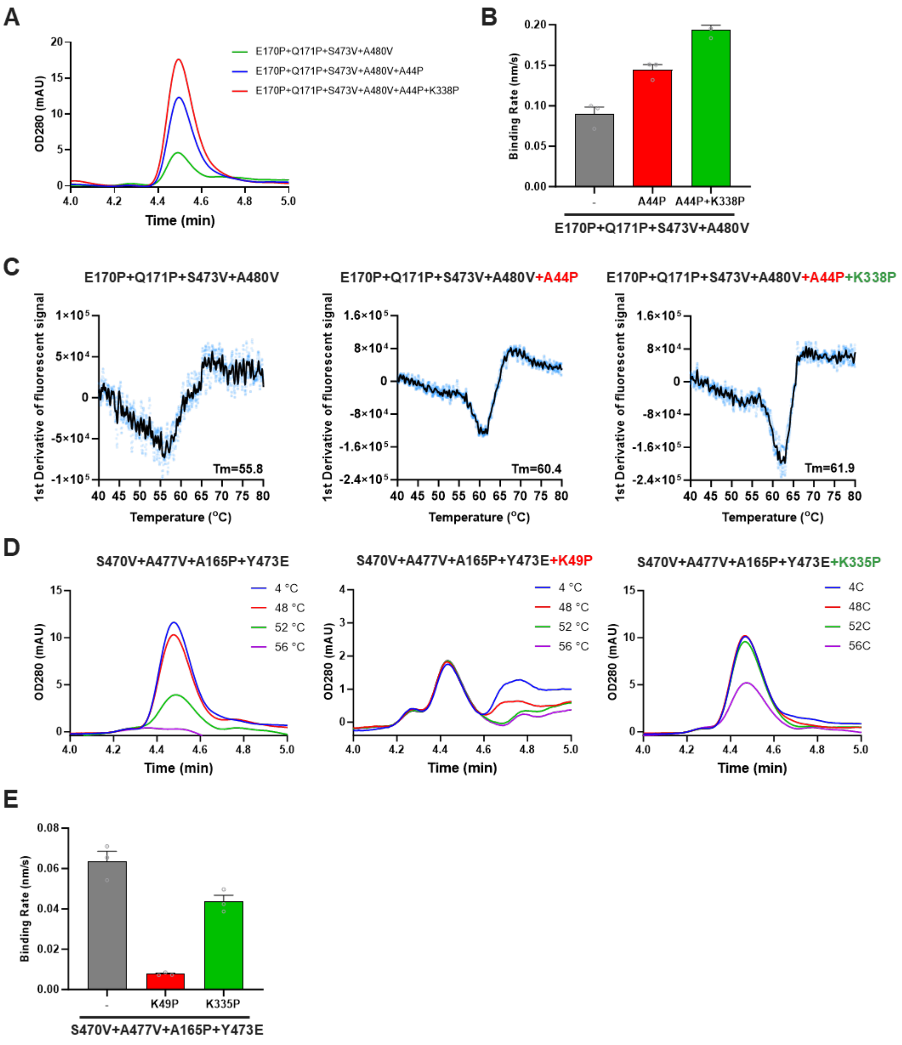

Supplementary Figure 7. Transfer of RV preF-stabilizing substitutions to RV1 and NiV F. (**A**) Analytical SEC on stabilized RV1 F variants in supernatant. RV1 substitutions A44P, E170P, Q171P, K338P, S473V, and A480V substitutions are the equivalents of the following substitutions in RV3, respectively: S41P, N167P, L168P, F335P, S470V, and S477V. (**B**) BLI using antibody 3X1 on indicated RV1 F variants in cell supernatant of (A). The initial slope, V0, at the start of binding is plotted as the average of three independent transfections; error bars represent the SD. (**C**) DSF on F variants in cell supernatant of (A). (**D**) Heat-SEC on stabilized NiV F variants. NiV substitutions K49P, K167P, K335P, S470V, and A477V are the equivalents of the following substitutions in RV3, respectively: S41P, N167P, F335P, S470V, and S477V. (**E**) BLI using antibody 5B3 on indicated NiV F variants in cell supernatant of (D). The initial slope, V0, at the start of binding is plotted as the average of three independent transfections; error bars represent the SD.

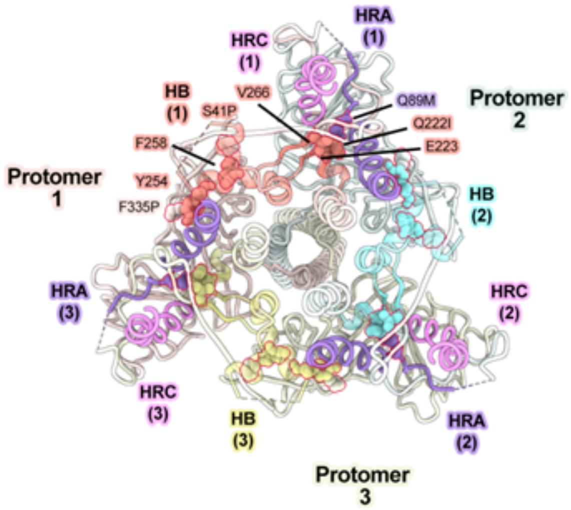

Supplementary Figure 8. Slice-through of the RV3 F head domain to visualize the band of stabilizing substitutions as viewed from the top. The structure is shown as ribbon representation with different structural elements highlighted as indicated in the figure.

Wildtype:

```
MPISILLIITTMIMASHCQIDITKLQHVGVLVNSPKGMKISQNFETRYLILSLIPKIEDSNSCGDQQIKQYKRLLDRLIIPLYDGL
RLQKDVIVTNQESNENTDPRTERFFGGVIGTIALGVATSAQITAAVALVEAKQARSDIEKLKEAIRDTNKAVQSVQSSVGNLIVAI
KSVQDYVNKEIVPSIARLGCEAAGLQLGIALTQHYSELTNIFGDNIGSLQEKGIKLQGIASLYRTNITEIFTTSTVDKYDIYDLLF
TESIKVRVIDVDLNDYSITLQVRLPLLTRLLNTQIYKVDSISYNIQNREWYIPLPSHIMTKGAFLGGADVKECIEAFSSYICPSDP
GFVLNHEMESCLSGNISQCPRTTVTSDIVPRYAFVNGGVVANCITTTCTCNGIGNRINQPPDQGVKIITHKECNTIGINGMLFNTN
KEGTLAFYTPDDITLNNSVALDPIDISIELNKAKSDLEESKEWIRRSNQKLDSIGGSEPEA
```

OnlyEcto2P:

```
MPISILLIITTMIMASHCQIDITKLQHVGVLVNSPKGMKIPQNFETRYLILSLIPKIEDSNSCGDQQIKQYKRLLDRLIIPLYDGL
RLMKDVIVTNQESNENTDPRTERFFGGVIGTIALGVATSAQITAAVALVEAKQARSDIEKLKEAIRDTNKAVQSVQSSVGPPIVAI
KSVQDYVNKEIVPSIARLGCEAAGLQLGIALTQHYSELTNIFGDNIGSLIEKGIKLQGIASLYRTNITEIFTTSTVDKYDIYDLLF
TESIKVRVIDVDLNDYSITLQVRLPLLTRLLNTQIYKVDSISYNIQNREWYIPLPSHIMTKGAFLGGADVKECIEAPSSYICPSDP
GFVLNHEMESCLSGNISQCPRTTVTSDIVPRYAFVNGGVVANCITTTCTCNGIGNRINQPPDQGVKIITHKECNTIGINGMLFNTN
KEGTLAFYTPDDITLNNSVALNPIDISIELNKAKSDLEEVKEWIRRVNQKLDSIGSGEPEA
```

OnlyEcto:

```
MPISILLIITTMIMASHCQIDITKLQHVGVLVNSPKGMKIPQNFETRYLILSLIPKIEDSNSCGDQQIKQYKRLLDRLIIPLYDGL
RLMKDVIVTNQESNENTDPRTERFFGGVIGTIALGVATSAQITAAVALVEAKQARSDIEKLKEAIRDTNKAVQSVQSSVGNPIVAI
KSVQDYVNKEIVPSIARLGCEAAGLQLGIALTQHYSELTNIFGDNIGSLIEKGIKLQGIASLYRTNITEIFTTSTVDKYDIYDLLF
TESIKVRVIDVDLNDYSITLQVRLPLLTRLLNTQIYKVDSISYNIQNREWYIPLPSHIMTKGAFLGGADVKECIEAFSSYICPSDP
GFVLNHEMESCLSGNISQCPRTTVTSDIVPRYAFVNGGVVANCITTTCTCNGIGNRINQPPDQGVKIITHKECNTIGINGMLFNTN
KEGTLAFYTPDDITLNNSVALDPIDISIELNKAKSDLEEVKEWIRRVNQKLDSIG
```

Supplementary Figure 9. Amino acid sequences of RV3 F wildtype, OnlyEcto2P and OnlyEcto. Stabilizing substitutions shared between OnlyEcto2P and OnlyEcto are highlighted in green, stabilizing substitutions unique to OnlyEcto2P are in magenta, and the linker/C-tag is highlighted in blue.

Supplementary Table 1

**EM DATA COLLECTION**	
Microscope (FEI)	Titan Krios
Voltage (kV)	300
Detector	Gatan K3
Pixel size (Å/pix)	0.81
Exposure rate (e^-/pix/s)	10
Frames per exposure	40
Exposure (e^-/Å^2)	70
Defocus range (μm)	1.5-2.5
Tilt angle (degrees,°)	30
Micrographs collected	2,061
Micrographs used	1,690
Particles extracted	488,241
Automation software	SerialEM
**Complex Composition**	**RV3 preF + VHH 4C06**
**FINAL 3D RECONSTRUCTION STATISTICS**	
Particles	193,265
Symmetry	C3
Map sharpening B-factor	-106
Resolution at FSC...	
Unmasked: 0.5 (Å)	3.3
Masked: 0.5 (Å)	3.2
Unmasked: 0.143 (Å)	3.0
Masked: 0.143 (Å)	3.0
**MODEL REFINEMENT AND VALIDATION STATISTICS**	
Composition	
Amino Acids (#)	1701
Ligands (Type: #)	0
RMSD Bonds	
Length [Å] (# > 4s)	0.009 (0)
Angles [°] (# > 4s)	1.04 (0)
Ramachandran plot	
Outliers (%)	0.00
Allowed (%)	3.70
Favored (%)	96.30
Rotamer outliers (%)	0.00
Average B-factors	
Amino acids	17.35
C-β outliers (%)	0.00
CaBLAM outliers (%)	0.91
CC (mask)	0.79
MolProbity score	1.63
Clash score	6.89
EMRinger score	3.11
