# Supplementary material for: Universal paramyxovirus vaccine design by stabilizing regions involved in structural transformation of the fusion protein
Source: Nat Commun. 2024 May 31;15:4629. doi: 10.1038/s41467-024-48059-w (PMC11143371; doi:10.1038/s41467-024-48059-w)
Supplement: Supplementary file 3 — Reporting Summary [file 41467_2024_48059_MOESM3_ESM.pdf]

# nature portfolio

Corresponding author(s): Mark Bakkers

Last updated by author(s): 03/28/2024



# Reporting Summary

Nature Portfolio wishes to improve the reproducibility of the work that we publish. This form provides structure for consistency and transparency in reporting. For further information on Nature Portfolio policies, see our Editorial Policies and the Editorial Policy Checklist.

## Statistics

For all statistical analyses, confirm that the following items are present in the figure legend, table legend, main text, or Methods section.

| n/a | Confirmed | |
|---|---|---|
| ☐ | ☒ | The exact sample size (*n*) for each experimental group/condition, given as a discrete number and unit of measurement |
| ☐ | ☒ | A statement on whether measurements were taken from distinct samples or whether the same sample was measured repeatedly |
| ☐ | ☒ | The statistical test(s) used AND whether they are one- or two-sided<br>*Only common tests should be described solely by name; describe more complex techniques in the Methods section.* |
| ☐ | ☒ | A description of all covariates tested |
| ☐ | ☒ | A description of any assumptions or corrections, such as tests of normality and adjustment for multiple comparisons |
| ☐ | ☒ | A full description of the statistical parameters including central tendency (e.g. means) or other basic estimates (e.g. regression coefficient) AND variation (e.g. standard deviation) or associated estimates of uncertainty (e.g. confidence intervals) |
| ☐ | ☒ | For null hypothesis testing, the test statistic (e.g. *F*, *t*, *r*) with confidence intervals, effect sizes, degrees of freedom and *P* value noted<br>*Give P values as exact values whenever suitable.* |
| ☒ | ☐ | For Bayesian analysis, information on the choice of priors and Markov chain Monte Carlo settings |
| ☒ | ☐ | For hierarchical and complex designs, identification of the appropriate level for tests and full reporting of outcomes |
| ☒ | ☐ | Estimates of effect sizes (e.g. Cohen's *d*, Pearson's *r*), indicating how they were calculated |

*Our web collection on statistics for biologists contains articles on many of the points above.*

## Software and code

Policy information about availability of computer code

| | |
|---|---|
| Data collection | Flow cytometry data was collected with FACSDiva v.9.0<br>SEC-MALS data were collected using Chromeleon 7.2.8.0<br>DSF data was collected with QuantStudio Real-Time PCR system software |
| Data analysis | ELISA and VNA (PRNT) titers were calculated with in-house custom made R scripts (R version 3.4.3) based on Gen5™ Data Analysis Software<br>Graph Pad Prism version 10<br>SEC-MALS data were analyzed using Chromeleon 7.2.8.0 and Astra 8.0.0.19 (Wyatt)<br>Statistical analyses for preclinical  experiments were performed using SAS version 9.4 (SAS Institute, Inc., Cary, NC, USA)<br>BLI data was analyzed using FortéBio Data Analysis 12.0 software (FortéBio)<br>Flow cytometry data was analyzed with FlowJo software v.10.8.1.<br>C3 map was processed using the DeepEMhancer tool within Cosmic2 Gateway<br>Motion correction, CTF-estimation, blob particle picking, and particle extraction was performed in cryoSPARC Live v3.2.0<br>Cryo-EM structure was build using ChimeraX and refined iteratively using Phenix, Coot, and ISOLDE. |

For manuscripts utilizing custom algorithms or software that are central to the research but not yet described in published literature, software must be made available to editors and reviewers. We strongly encourage code deposition in a community repository (e.g. GitHub). See the Nature Portfolio guidelines for submitting code & software for further information.

## Data

Policy information about availability of data

All manuscripts must include a data availability statement. This statement should provide the following information, where applicable:

- Accession codes, unique identifiers, or web links for publicly available datasets
- A description of any restrictions on data availability
- For clinical datasets or third party data, please ensure that the statement adheres to our policy

All of the final data has been included in main figures or supplementary information, the structure map will be deposited in available online databases.The raw data that support the findings of this study are available from the corresponding author upon reasonable request.

## Research involving human participants, their data, or biological material

Policy information about studies with human participants or human data. See also policy information about sex, gender (identity/presentation), and sexual orientation and race, ethnicity and racism.

| | |
|---|---|
| Reporting on sex and gender | No human research was performed |
| Reporting on race, ethnicity, or other socially relevant groupings | No human research was performed |
| Population characteristics | No human research was performed |
| Recruitment | No human research was performed |
| Ethics oversight | No human research was performed |

Note that full information on the approval of the study protocol must also be provided in the manuscript.

# Field-specific reporting

Please select the one below that is the best fit for your research. If you are not sure, read the appropriate sections before making your selection.

☒ Life sciences ☐ Behavioural & social sciences ☐ Ecological, evolutionary & environmental sciences

For a reference copy of the document with all sections, see nature.com/documents/nr-reporting-summary-flat.pdf

# Life sciences study design

All studies must disclose on these points even when the disclosure is negative.

| | |
|---|---|
| Sample size | Sample size of the different mouse and cotton rats studies described in the manuscript are determined based on power calculations. Historical data for the investigated or similar vaccines were used to guide statistical powering. Sample sizes were calculated using SAS version 9.4 (SAS Institute, Inc., Cary, NC, USA) |
| Data exclusions | All data is included and shown |
| Replication | binding and neutralization antibody titers were always measured as technical duplicates.<br>Screen for RV3 preF-stabilizing substitutions and related experiments were performed in biological triplicate.<br>Fusion assay was performed in biological duplicate<br>All attempts at reproduction were successful. |
| Randomization | Mice and cotton rats were randomly assigned to groups. For all other experiments samples or wells, were allocated randomly. |
| Blinding | Investigators were not blinded during ELISA and VNA data collection, but serum samples were randomly mixed onto the measurement plates for ELISA measurements to minimizing group specific biases in data collection. Investigators were blinded during cryo-EM analysis. Investigators were not blinded during Octet, DSF, SEC and FACS experiments, since there was no preference towards use of any of the stabilizing substitutions or vaccine candidates and hence there was no scope for bias in unblinded testing. |

# Reporting for specific materials, systems and methods

We require information from authors about some types of materials, experimental systems and methods used in many studies. Here, indicate whether each material, system or method listed is relevant to your study. If you are not sure if a list item applies to your research, read the appropriate section before selecting a response.

## Materials & experimental systems

| n/a | Involved in the study |
| --- | --- |
| | ☒ Antibodies |
| | ☒ Eukaryotic cell lines |
| ☒ | Palaeontology and archaeology |
| | ☒ Animals and other organisms |
| ☒ | Clinical data |
| ☒ | Dual use research of concern |
| ☒ | Plants |

## Methods

| n/a | Involved in the study |
| --- | --- |
| ☒ | ChIP-seq |
| | ☒ Flow cytometry |
| ☒ | MRI-based neuroimaging |

# Antibodies

Antibodies used

PIA174 (Stewart-Jones et al. PNAS 2018); produced at U-protein Express
VHH 4C06 and VHH 4C03 (Johnson et al, currently in review at Nat. Comm) were produced in-house.
Goat anti mouse IgG-HRP (Biorad Cat172-1011)

Validation

PIA174 was validated in: doi: 10.1073/pnas.1811980115
VHH 4C06 and VHH 4C03 were validated by Jonhson et al, which is currently in review at Nat. Comm
Goat anti mouse IgG-HRP is commercially available. Validation information is available on the manufacturers' websites.

# Eukaryotic cell lines

Policy information about cell lines and Sex and Gender in Research

Cell line source(s)

Human embryonic kidney cell line expi293F (Thermo Fisher; A14527)
Human Embryonic Kidney (HEK) 293 cells (ATCC; CRL-1573)
VERO cells were received from the WHO (WHO10-87; 880101), a working cell bank was prepared directly.

Authentication

Authentication was performed by supplier

Mycoplasma contamination

All cell lines were tested as being mycoplasma negative by supplier.

Commonly misidentified lines
(See ICLAC register)

No commonly misidentified cell lines were used in this study

# Animals and other research organisms

Policy information about studies involving animals; ARRIVE guidelines recommended for reporting animal research, and Sex and Gender in Research

Laboratory animals

Mouse studies: BALB/c mice, female, 6- to 8-weeks-old at the start of the study; cotton rat studies: female, 6- to 8-weeks old at the start of the study. Data on housing conditions are mentioned in the Methods section.

Wild animals

The study did not involve wild animals.

Reporting on sex

Studies were only performed in female animals.  For a careful comparison of induced immune responses between (combinations of) different vaccine candidates, as was the main objective of studies described in the current manuscript, it is important to keep the groups of experimental animals as homogeneous as possible. Therefore, experimental groups (in rodent experiments) were mostly comprised of inbred animals, from the same strain and age, as well as from the same sex. By reducing the variation, group sizes can be smaller, requiring fewer animals to address a specific research question. In the human population, more heterogenous immune responses can be expected. In addition to sex differences, many other factors (eg, genetic background,  pre-exposure history, underlying health conditions, and age) contribute to this heterogeneity. Therefore, in later stages of vaccine development, human testing in a heterogenous population comprised of both sexes is part of the vaccine development program.

Field-collected samples

Study did not involve samples collected from the field.

Ethics oversight

Mouse studies were conducted at Janssen Vaccines and Prevention B.V. according to the Dutch Animal Experimentation Act and the Guidelines on the Protection of Animals for scientific purposes by the Council of the European Committee after approval by the Centrale Commissie Dierproeven and the Dier Experimenten Commissie of Janssen Vaccines and Prevention B.V.
Cotton rat studies were conducted at Sigmovir Biosystems, Inc. by permission of the Institutional Animal Care and Use Committee (IACUC) of Sigmovir Biosystems, Inc.

Note that full information on the approval of the study protocol must also be provided in the manuscript.

## Plants

Seed stocks

*Report on the source of all seed stocks or other plant material used. If applicable, state the seed stock centre and catalogue number. If plant specimens were collected from the field, describe the collection location, date and sampling procedures.*

Novel plant genotypes

*Describe the methods by which all novel plant genotypes were produced. This includes those generated by transgenic approaches, gene editing, chemical/radiation-based mutagenesis and hybridization. For transgenic lines, describe the transformation method, the number of independent lines analyzed and the generation upon which experiments were performed. For gene-edited lines, describe the editor used, the endogenous sequence targeted for editing, the targeting guide RNA sequence (if applicable) and how the editor was applied.*

Authentication

*Describe any authentication procedures for each seed stock used or novel genotype generated. Describe any experiments used to assess the effect of a mutation and, where applicable, how potential secondary effects (e.g. second site T-DNA insertions, mosiacism, off-target gene editing) were examined.*

## Flow Cytometry

### Plots

Confirm that:

☐ The axis labels state the marker and fluorochrome used (e.g. CD4-FITC).

☐ The axis scales are clearly visible. Include numbers along axes only for bottom left plot of group (a 'group' is an analysis of identical markers).

☐ All plots are contour plots with outliers or pseudocolor plots.

☐ A numerical value for number of cells or percentage (with statistics) is provided.

### Methodology

Sample preparation

*Describe the sample preparation, detailing the biological source of the cells and any tissue processing steps used.*

Instrument

*Identify the instrument used for data collection, specifying make and model number.*

Software

*Describe the software used to collect and analyze the flow cytometry data. For custom code that has been deposited into a community repository, provide accession details.*

Cell population abundance

*Describe the abundance of the relevant cell populations within post-sort fractions, providing details on the purity of the samples and how it was determined.*

Gating strategy

*Describe the gating strategy used for all relevant experiments, specifying the preliminary FSC/SSC gates of the starting cell population, indicating where boundaries between "positive" and "negative" staining cell populations are defined.*

☐ Tick this box to confirm that a figure exemplifying the gating strategy is provided in the Supplementary Information.
